# Negative Affect, Sensation Seeking, and Adolescent Substance Use Development: The Moderating Role of Executive Function

**DOI:** 10.1007/s10964-024-02065-9

**Published:** 2024-08-10

**Authors:** Ann Folker, Kristin M. Peviani, Kirby Deater-Deckard, Warren K. Bickel, Laurence Steinberg, Brooks Casas, Jungmeen Kim-Spoon

**Affiliations:** 1 Department of Psychological and Brain Sciences, University of Massachusetts, Amherst, MA USA; 2 Department of Psychology, Virginia Tech, Blacksburg, VA USA; 3 Helsinki Collegium for Advanced Studies, University of Helsinki, Helsinki, Finland; 4 Fralin Biomedical Research Institute at VTC, Roanoke, VA USA; 5 Department of Psychology and Neuroscience, Temple University, Philadelphia, PA USA

**Keywords:** Substance use, Executive function, Negative affect, Sensation seeking, Adolescence

## Abstract

It is unknown how the Addictions Neuroclinical Assessment markers—negative affect, sensation seeking, and executive function—contribute to substance use development. This study examined whether associations of negative affect and sensation seeking with substance use vary by executive function. Participants were 167 adolescents (47% female) who participated annually for four years (*M*_*age*_ = 14.07, *SD*_*age*_ = 0.54 at Time 1). There were within-person bidirectional associations between higher negative affect and higher substance use for adolescents with lower executive function. Adolescents with higher sensation seeking at age 14 exhibited increasing substance use trajectories from age 14 to 17, regardless of executive function level. Negative affect and substance use influence each other within individuals, whereas sensation seeking predicts substance use between individuals.

## Introduction

Adolescence is a developmental period characterized by rapid biological, social, and emotional changes including increased autonomy, more time spent with peers, and changes in relationships with parents and other caregivers (Steinberg, 2014). Substance use is typically initiated during this time and is associated with an increased likelihood of developing substance use disorders later in life (Gray & Squeglia, 2018). In the United States, 15% of adolescents meet criteria for alcohol abuse, and 16% meet the criteria for drug abuse by age 18 (Swendsen et al., 2012). The effects of substance use during adolescence can be long lasting in part due to neurotoxin exposure which can have detrimental effects on cognition and emotional functioning and increase potential for substance use disorders and addiction in adulthood (Brown et al., 2008). From a neurobiological perspective, during adolescence, brain regions responsible for lower order emotion processing functions mature sooner than frontal regions associated with higher order cognitive functioning (Casey et al., 2008). The imbalance between these emotional reactivity and cognitive control systems renders adolescents particularly vulnerable to risk-taking behaviors such as substance use (Casey et al., 2008; Steinberg, 2008). Relatedly, the Addictions Neuroclinical Assessment model (Kwako et al., 2018) offers a more specific dimensional approach to understanding substance use by outlining three domains implicated in addiction that overlap with three of the six Research Domain Criteria (RDoC; Insel, 2010). These domains are (1) incentive salience (feelings of reward from stimuli associated with substances), (2) negative affect (emotional state characterized by unpleasant or lack of feelings), and (3) executive function (higher-order cognitive abilities such as inhibition, set shifting, and working memory; Kwako et al., 2018). The present study applied the Addictions Neuroclinical Assessment model to understand the transactional nature of risk and protective factors for substance use development during adolescence, viewing the heterogeneity of substance use as a function of the interface between emotional risk (incentive salience and negative affect) and cognitive protective (executive function) factors. In doing so, sensation seeking in adolescents was assessed as a proxy for incentive salience, given the relevance of sensation seeking in reflecting adolescents’ approach to substance use (i.e., sensation seeking is correlated with reward sensitivity; Harden et al., 2018). Prior research has noted moderate to strong associations between reward sensitivity and sensation seeking in adolescents (Scott-Parker et al., 2012). Additionally, neuroimaging work provides evidence for associations between heightened neural response to rewards and sensation seeking in late adolescence (Hawes et al., 2017), thus providing rationale for using sensation seeking in the present study.

In the current literature, the three domains of the Addictions Neuroclinical Assessment are implicated in substance use progression in various ways. High sensation seeking contributes to vulnerability for substance use (LaSpada et al., 2020). For individuals with high negative affect, substance use may be a coping mechanism to relieve feelings of negativity (Magee & Connell, 2021). The evidence for direct effects of executive function on substance use is mixed, and may vary with effect size (Aytaclar et al., 1999). More convincing evidence suggests that executive function interacts with other emotion or motivation variables such as negative affect and sensation seeking to predict substance use (Kim-Spoon et al., 2017). For example, associations between substance use, frustration, and reward sensitivity are weaker for adolescents with high cognitive control, compared to those with low cognitive control (Kim-Spoon et al., 2016), highlighting the regulatory role of executive function.

To better understand the etiology of substance use, prior work has proposed internalizing and externalizing pathways to substance use problems in adolescence, suggesting two distinct categories of adolescents who may be especially vulnerable to substance use. The externalizing pathway underscores the role of delinquent behavior, which is often driven by sensation seeking (Edwards et al., 2016), whereas the internalizing pathway emphasizes negative affect and self-medication as potential factors contributing to substance use (Hussong et al., 2011). Importantly, these theorized pathways may operate at both within- and between-person levels, such that having higher negative affect or sensation seeking than usual for an individual may lead to increased substance use (within-person process). Additionally, those with higher negative affect or sensation seeking on average may use substances more than those with lower negative affect (between-person process). Although these processes have been tested primarily at the between-person level, a few studies have also examined the associations between negative affect or sensation seeking and substance use at both the between- and within-person level. In a sample of Chinese late adolescents, there were positive bi-directional year-to-year associations between sensation seeking and substance use (combined cigarette and alcohol use) at the within-person level as well as a positive association between sensation seeking change and substance use change over three years at the between-person level (Shen et al., 2023). Similarly, another study found both between- and within- person associations between sensation seeking and alcohol use in a national sample of adolescents from the United States (Waddell & Sasser, 2022). Conversely, in another sample, only within-person associations between sensation seeking and binge drinking were observed (Waddell & Chassin, 2023). Given these mixed findings, it is important to further investigate the associations between sensation seeking and substance use at both within-person and between-person levels. In a study of U.S. late adolescents, albeit not directly assessing negative affect, there were positive bi-directional year-to-year associations between depression and alcohol coping motives at the within-person level, as well as a positive concurrent association between depression and alcohol coping motives at the between-person level (Colder et al., 2019). In this study, depression was not associated with alcohol consumption at either the between-person or within-person level.

Understanding the roles of negative affect and sensation seeking on substance use at the within-person level is theoretically crucial for two reasons: First, the motivational processes (i.e., negative affect and sensation seeking) of the Addictions Neuroclinical Assessment model are within-person determinants of the pathways to substance use progression and addiction (e.g., elevated negative affect/sensation seeking predicts future engagement in substance use). As such, modeling within-person processes is arguably the most relevant for better understanding developmental processes whereby negative affect and sensation seeking contribute to substance use progression. Second, between- and within-person effects may differ, and thus disaggregating their distinct effects is important. For example, prior studies focusing only on between-person associations demonstrated that adolescents with higher levels of negative affect are more likely to engage in substance use to cope with negative affect, and substance use is associated with higher negative affect on average (Mason et al., 2009; Measelle et al., 2006). In contrast, a recent meta-analysis summarizing within-person negative affect- substance use associations suggested non-significant effects of negative affect on substance use (measured via alcohol use) when daily diary approaches were utilized (Dora et al., 2023). Yet, traditional statistical methods blend between- and within-person effects, making results difficult to interpret (Berry & Willoughby, 2017). Better understanding of how negative affect and sensation seeking relate to adolescent substance use at both within- and between-person levels will aid in preventive interventions for problematic substance use among youth and adolescents by clarifying the target population (i.e., between-person prediction) and identifying risk factors for substance use and progression toward addiction (i.e., within-person prediction).

## Current Study

It is unknown how negative affect and sensation seeking relate to adolescent substance use at both the within- and between-person levels. Further, the role of executive function in these associations is understudied. The present study examined developmental processes through which the three key domains of the Addictions Neuroclinical Assessment model (negative affect, sensation seeking, executive function) contribute to substance use progression. Longitudinal bidirectional (i.e., reciprocal) associations between negative affect and substance use, as well as sensation seeking and substance use across adolescence at both the within- and between-person levels were examined. Further, the present study investigated the moderating role of executive function in these associations with the expectation that the substance use risks driven by negative affect and sensation seeking may vary by level of executive function such that higher negative affect and sensation seeking would be associated with greater substance use longitudinally, but their effects would be reduced at higher levels of executive function (Hypothesis 1). Given the lack of research on within-person associations between the Addictions Neuroclinical Assessment dimensions and substance use, the present study did not have specific hypotheses regarding whether the expected associations would manifest at either the between- or within-person level or both.

## Methods

### Participants

Participants were 167 adolescents (53% male) who participated in a longitudinal study annually for four years. Adolescents were 13−14 years of age at Time 1 (*n* = 167, *M* = 14.07, *SD* = 0.54), 14−15 years of age at Time 2 (*n* = 157, *M* = 15.05, *SD* = 0.54), 15-16 years of age at Time 3 (*n* = 150, *M* = 16.08, *SD* = 0.55), and 16-17 years of age at Time 4 (*n* = 148, *M* = 17.02, *SD* = 0.55). The majority of adolescents identified as White (78.4%), with the rest identifying as another race. The median household income of the sample was $35,000–$49,999. Using an income-to-needs (ITN) ratio, about 50% of the sample was considered poor/near poor. At Time 1, 157 adolescents participated. At Time 2, 10 adolescents were added to offset annual attrition for a final sample of 167 (150 at Time 2, 147 at Time 3, and 150 at Time 4). Across all four years, 24 adolescents did not participate at all four time points for reasons including: declined participation (*n* = 17), lost contact (*n* = 5), and ineligibility for tasks (*n* = 2). Rate of participation was not significantly predicted by income, sex, race or study variables (*p*s ≥ 0.068).

### Procedures

Adolescents were recruited via flyers, letters, and e-mail. Those who indicated interest in the study were contacted by research assistants who described the study to them. Data collection took place at the university, and all adolescents provided written assent and parents completed written consent prior to participation. Behavioral, questionnaire and neuroimaging data were collected as a part of the larger study, but only behavioral and questionnaire data were reported herein. Due to the protocol including neuroimaging procedures, adolescents were ineligible to participate if they had contraindications to magnetic resonance imaging. Adolescents received monetary compensation for participation. Procedures were approved by the University’s institutional review board.

### Measures

#### Negative Affect

Adolescent self-report on the negative affect subscale of the Positive and Negative Affect Schedule (PANAS; Watson et al., 1988; Laurent et al., 1999) was used to assess adolescent negative affect at each time point. Adolescents responded on a 5-point scale (1 = *very slightly or not at all* to 5 = *extremely*) to questions asking the extent to which they have felt certain emotions over the past few weeks (i.e., irritable, active, guilty). An overall negative affect score was created by averaging the adolescents’ responses, with a higher score indicating higher negative affect. Reliability for this scale in the sample was acceptable across the four timepoints (α = 0.61–0.75).

#### Sensation Seeking

Sensation seeking was measured using a subset of six items from the adolescent-report of the Sensation Seeking Scale (SSS; Zuckerman et al., 1978). Adolescents responded to six true/false items asking about adventure seeking, disinhibition, experience seeking, and boredom susceptibility (i.e., “I like doing things just for the thrill of it”). An average sensation seeking score was computed by averaging responses across the six items. Higher scores indicated greater sensation seeking/incentive salience. Reliability was acceptable across the four timepoints (α = 0.67–0.69). The full 19-item SSS includes items related to impulsivity (e.g., “I often do things on impulse”). Six items specifically related to sensation seeking were utilized to distinguish between sensation seeking and impulsivity.

#### Substance Use

Adolescents self-reported the frequency of typical use of cigarettes, alcohol, and cannabis at each annual assessment. A composite substance use score was created by averaging the frequency of use across three most commonly used substances (i.e., cigarettes, alcohol, cannabis). The decision to use the poly-substance use outcome was based on the literature demonstrating that use of multiple substances is common during adolescence (see Halladay et al., 2020 for review). Response options ranged from 1 = *never used* to 6 = *usually every day*. Reliability was acceptable across the four timepoints (α = 0.81–0.88).

#### Executive Function

A composite executive function measure was computed using an average of adolescents’ inhibitory control, set shifting, and working memory behavioral assessment scores across the four timepoints. First, the Multi-Source Interference Task (MSIT; Bush et al., 2003) was used to assess inhibitory control. In this task, adolescents were presented with three digits and instructed to identify the target number that was different from the others. The target’s identity was congruent with the target’s relative position for trials in the *neutral* condition, whereas the target’s identity did not match its relative position in the *interference* condition. The standard deviation of the reaction time for correct responses in interference trials was used as an indicator of inhibitory control (MacDonald et al., 2012). The value was reverse scored, such that higher values indicated better inhibitory control. Second, the Wisconsin Card Sorting Task (WCST; Heaton & Staff, 2003) was used to assess set shifting. The number of perseverative errors (mistakes using the incorrect matching rule continuously) was used to indicate difficulty with set shifting. This score was multiplied by −1 such that higher values indicated better set shifting. Finally, working memory was assessed using backward digit span from the Stanford Binet Intelligence Test (Thorndike et al., 1986). In this task, adolescents were presented with a string of digits of increasing length and instructed to repeat the sequence in the same or reverse order. A total score was computed by subtracting the total number of failed items from the highest administered span. Thus, higher scores indicated better working memory. Standardized scores of inhibitory control, set shifting, and working memory were averaged to calculate the executive function composite scores for each time point.

### Data Analysis Plan

**Fig. 1:**
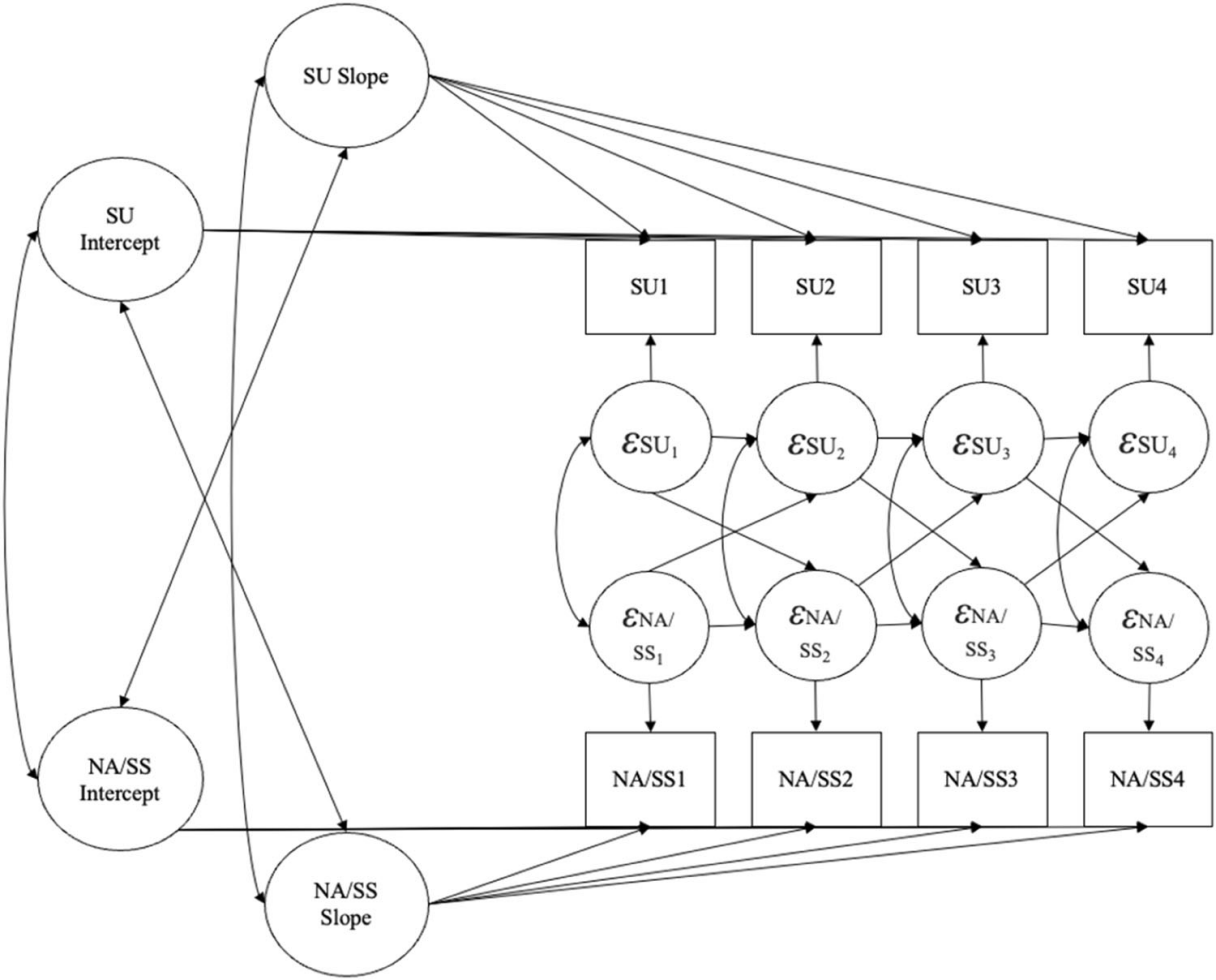
Path Diagram of Bivariate Latent Growth Curve Model with Structured Residuals (LCM-SR) for the Associations between Negative Affect/Sensation Seeking and Substance Use. Note. NA = negative affect; SS = sensation seeking; SU = substance use

Latent curve modeling with structured residuals (LCM-SR; Curran et al., 2014) was used to estimate the bidirectional associations between negative affect and substance use and sensation seeking and substance use at the between- and within-person level. Executive function was split into “extreme low” (lower 25%) and “high” (upper 75%) groups and examined as a moderator of these bidirectional associations. Figure 1 presents the path diagram for the models. Analyses were conducted in *Mplus* (Muthén & Muthén, 1998–2018). Model fit was assessed using the comparative fit index (CFI), Tucker Lewis Index (TLI), Akaike information criterion (AIC), Bayesian information criterion (BIC), Root Mean Square Error of Approximation (RMSEA), Standardized Root Mean Squared Residual (SRMR), and model chi-square (χ^2^). As recommended by Little (2013), acceptable model fit is considered: CFI ≥ 0.90 and TLI ≥ 0.90. For RMSEA, values should be below 0.1 (Kenny et al., 2015), and SRMR below 0.06 (Hu & Bentler, 1999). Lower AIC and BIC values indicate better fit. To compare nested models, chi-square differences were examined, with a significant ∆χ^2^ statistic (*p* < 0.05) indicating significant improvement in model fit, as well as differences in CFI > 0.01 indicating significant improvement in model fit (Cheung & Rensvold, 2002). A model building approach was taken as follows: First, univariate growth curve models were estimated for negative affect, sensation seeking, and substance use. These models were estimated first with intercept only (slope constrained to 0), then with a linear slope, and lastly with latent basis growth (i.e., first and last paths constrained to 0 and 1, respectively, and the second and third paths allowed to vary freely; Grimm et al., 2017) to determine the optimal shape of growth for each variable. Second, bivariate growth curve models between negative affect and substance use and sensation seeking and substance use were estimated using the whole sample. In these models, covariances between the intercept and slope of each variable were estimated. Third, structured residuals were added to the bivariate growth curve models, with autoregressive and cross-lagged residual paths. The cross-lagged paths were first constrained such that they were equal across time, and then allowed to vary freely over time to test whether the between-construct cross-lagged paths changed across ages. Chi-square difference tests were utilized to determine whether model fit improved significantly between the model with constrained cross-paths and free cross-paths. Lastly, the grand mean of adolescent executive function across the four timepoints was examined as a moderator using two-group bivariate growth curve models that compared high vs. low executive function groups. The missing data pattern was determined to be missing completely at random based on Little’s MCAR test (χ^2^ = 128.86, *df* = 128, *p* = 0.462). Full information maximum likelihood (FIML) was used to allow all available data to be included regardless of the pattern of missingness.

## Results

Table 1 presents descriptive statistics and bivariate correlations between study variables. Table 2 presents the model fit indices for the univariate growth curve models. For substance use and sensation seeking, the chi-square difference test and change in CFI indicated that the linear growth models fit the data best. For negative affect, the chi-square difference test indicated marginal improvement in model fit for the linear model, compared to the intercept-only model (*p* = 0.070). Based on improvement in other model fit indices (e.g., ∆ CFI > 0.01, decrease in AIC), and in alignment with the substance use and sensation seeking models, the linear model was retained as the final model.

**Table 1:** Descriptive Statistics and Correlations among Negative Affect, Sensation Seeking, Substance Use and Executive Function

	1	2	3	4	5	6	7	8	9	10	11	12	13
1. NA T1	–												
2. NA T2	0.615***	–											
3. NA T3	0.558***	0.620***	–										
4. NA T4	0.380***	0.377***	0.352***	–									
5. SS T1	0.180*	0.118	0.093	0.078	–								
6. SS T2	0.112	0.078	0.092	0.097	0.655***	–							
7. SS T3	0.161	−0.049	−0.078	0.079	0.534***	0.694***	–						
8. SS T4	0.132	−0.057	0.020	−0.025	0.552***	0.587***	0.722***	–					
9. SU T1	0.142	0.112	0.165	0.047	0.297***	0.116	0.057	0.064	–				
10. SU T2	0.141	0.196*	0.226**	0.093	0.300***	0.275***	0.155	0.120	0.701***	–			
11. SU T3	0.109	0.137	0.261**	0.128	0.315***	0.267**	0.238**	0.159	0.630***	0.769***	–		
12. SU T4	0.119	0.054	0.143	0.169*	0.326***	0.231**	0.242**	0.244**	0.502***	0.642***	0.796***	–	
13. EF	−0.122	0.003	−0.047	−0.114	−0.143	−0.132	−0.071	−0.078	−0.145	−0.160	−0.192*	−0.182*	–
*N*	157	149	143	141	154	149	143	141	157	150	147	149	167
*M*	1.891	1.974	1.966	1.997	0.601	0.574	0.596	0.588	1.217	1.371	1.611	1.978	−0.021
*SD*	0.711	0.623	0.651	0.708	0.269	0.281	0.272	0.264	0.388	0.582	0.702	0.965	0.616

*NA* negative affect, *SS* sensation seeking, *SU* substance use, *EF* executive function (grand mean across four Times), T1 = Time 1, T2 = Time 2, T3 = Time 3, T4 = Time 4

*p < 0.05, **p < 0.01, ***p < 0.001

**Table 2:** Model Fit for Univariate Growth Curve Models

	Intercept Only	Linear Slope^a^	Latent Basis
Negative Affect			
χ^2^ (*df*)	11.295 (8)	4.249 (5)	2.146 (3)
TLI	0.985	1.000	1.000
CFI	0.980	1.000	1.000
RMSEA	0.050	<0.001	<0.001
SRMR	0.124	0.055	0.016
AIC	1051.308	1050.263	1052.159
BIC	1070.007	1078.325	1086.457
∆χ^2^ (∆*df*)	–	7.046 (3)	2.103 (2)
Sensation Seeking			
χ^2^ (*df*)	22.216 (8)	13.357 (5)	17.923 (3)
TLI	0.961	0.964	0.892
CFI	0.949	0.970	0.946
RMSEA	0.103	0.100	0.173
SRMR	0.084	0.054	0.049
AIC	−115.636	−118.495	−109.928
BIC	−96.928	−90.433	−75.631
∆χ^2^ (∆df)	–	8.859 (3)*	4.566 (2)
Substance Use			
χ^2^ (*df*)	308.955 (8)	13.307 (5)	10.862 (3)
TLI	0.388	0.973	0.957
CFI	0.183	0.977	0.979
RMSEA	0.475	0.100	0.125
SRMR	0.504	0.076	0.078
AIC	1080.050	790.402	791.957
BIC	1098.758	818.464	826.254
∆χ^2^ (∆*df*)	–	295.648 (3)***	2.445 (2)

For negative affect the chi-square difference test between the linear and intercept-only models was not significant (*p* = 0.070). The linear growth model was retained as the final model because of improvement in other model fit indices

*TLI* Tucker-Lewis index, *CFI* Comparative Fit Index, *RMSEA* Root Mean Square Error of Approximation, *SRMR* Standardized Root Mean Squared Residual, *AIC* Akaike Information Criterion, *BIC* Bayesian Information Criterion

*p < 0.05, ***p < 0.001

^a^Final retained model

Table 3 presents the coefficients for each univariate model. Substance use had a significant, positive slope, indicating increasing substance use on average across adolescence. Negative affect and sensation seeking had non-significant means of slope factors, indicating non-significant systematic patterns of change as a whole group. Substance use and sensation seeking had significant variances of the slope factor, indicating significant individual differences in change rates across time. All three variables had significant variances of the intercept factor, indicating that there were significant individual differences in levels of substance use, sensation seeking, and negative affect at age 14.

**Table 3:** Growth Factors for Univariate Linear Growth Models

	Intercept		Slope	
Variable	Mean	Variance	Mean	Variance
Negative Affect	1.916***	0.296***	0.026	0.002
Sensation Seeking	0.598***	0.049***	−0.001	0.003**
Substance Use	1.210***	0.143***	0.225***	0.060***

All effects are unstandardized

**p < 0.01, ***p < 0.001

### Growth Curve Modeling of Executive Function

For executive function as the moderator, we used the grand mean of a given individual’s repeated measures as estimates of trait scores because they are averages over time (and situation) for each individual (e.g., Kim & Cicchetti, 2009). Consistent executive function (i.e., non-significant average change and variability) would further justify aggregating executive function scores across time by calculating within-person grand means across four time points to represent each individual’s general level of executive function. A linear latent growth curve model was estimated and revealed no significant change on average in executive function across the four years (*γ*_*10*_ = −0.001, *SE* = 0.02, *p* = 0.953), and no significant individual differences in this change (*τ*_*11*_ = 0.001, *SE* = 0.01, *p* = 0.813). The results indicated that executive function did not change over time, and there were no significant individual differences in the change of executive function across the four years. As such, individuals’ grand mean across the four waves was used as the grouping variable to test moderating effects in the analysis. The non-significant mean and variance of the slope factor was not surprising considering that the executive function composite was calculated by averaging the three component scores that were standardized within each assessment time (i.e., the mean of the individual component scores was zero).

### Bivariate Growth Curve Models

Table 4 presents model fit statistics for the bivariate growth curve models, following the model building approach outlined in the Data Analysis Plan. For both the negative affect and sensation seeking models, model fit did not significantly improve when cross-lags were allowed to vary over time compared to when they were constrained to be equal over time; thus, the models with constrained cross-lags were retained as the final models. Both models also included within-person auto-regressive paths that were constrained to be equal over time. For these models, sex (male/female), race (White/non-White), and income (ITN ratio) were tested as predictors of substance use. None were significantly associated with substance use and were therefore not included in the models as covariates. Models were also estimated with these covariates included, and there were no substantial changes in results. Table S1 presents results from models with sex, race, and income included.

**Table 4:** Model Fit for Bivariate Growth Curve Models with Substance Use

	Bivariate	Constrained cross-lags^a^	Free cross-lags
Negative Affect			
χ^2^ (*df*)	37.033 (22)	17.267 (16)	17.320 (13)
TLI	0.965	0.996	0.983
CFI	0.973	0.998	0.992
RMSEA	0.064	0.038	0.045
SRMR	0.062	0.060	0.041
AIC	1838.136	1830.370	1836.423
BIC	1906.732	1917.674	1933.081
∆χ^2^ (∆*df*)	–	19.766 (6)**	0.053 (3)
Sensation Seeking			
χ^2^ (*df*)	42.159 (22)	14.837 (16)	12.178 (12)
TLI	0.962	1.000	0.999
CFI	0.970	1.000	1.000
RMSEA	0.074	0.054	0.009
SRMR	0.055	0.058	0.025
AIC	652.316	636.994	642.335
BIC	720.912	724.298	742.111
∆χ^2^ (∆*df*)	–	27.322 (6)***	2.659 (4)

*TLI* Tucker-Lewis index, *CFI* Comparative Fit Index, *RMSEA* Root Mean Square Error of Approximation, *SRMR* Standardized Root Mean Squared Residual, *AIC* Akaike Information Criterion, *BIC* Bayesian Information Criterion

**p < 0.01, ***p < 0.001

^a^Final retained model

Table 5 presents between- and within-person associations for the extreme low (i.e., bottom 25%) and high (i.e., top 75%) executive function groups, and for the full sample. As a sensitivity analysis, we also examined the model with executive function split at the median (i.e., 50% low, 50% high). These results are presented in Table S2. The two-group negative affect-substance use model revealed significant within-person cross-lagged associations between negative affect and substance use for the low executive function group only, suggesting that higher negative affect was associated with subsequent increased substance use only for those with low executive function. This effect was significantly different than the effect for the high executive function group (Wald test *χ*^2^ = 9.207, *df* = 1, *p* = 0.002). Furthermore, there was a significant within-person level cross-lagged association from substance use to negative affect for the low executive function group, suggesting that higher substance use was associated with increased negative affect. This effect was significantly different than the effect for the high executive function group (Wald test *χ*^2^ = 7.307, *df* = 1, *p* = 0.007). There were no significant between-person associations in the negative affect-substance use model.

**Table 5:** Latent Growth Factor Correlations and Cross-Lagged Regression Coefficients between Negative Affect/Sensation Seeking and Substance Use at High and Low Executive Function

	High EF	Low EF	Full Sample
	*b (SE)*	*b (SE)*	*b (SE)*
Negative Affect			
Intercept Mean	1.874 (0.060)***	1.932 (0.109)***	1.929 (0.053)***
Intercept Variance	0.276 (0.078)***	0.431 (0.302)	0.278 (0.090)**
Slope Mean	0.028 (0.023)	0.055 (0.043)	0.027 (0.020)
Slope Variance	0.003 (0.017)	−0.007 (0.036)	−0.004 (0.018)
NA slope ↔ SU intercept	−0.009 (0.010)	0.045 (0.064)	−0.005 (0.013)
NA intercept ↔ SU slope	0.016 (0.017)	−0.001 (0.068)	0.003 (0.018)
NA intercept ↔ SU intercept	0.029 (0.027)	−0.074 (0.242)	0.035 (0.036)
NA slope ↔ SU slope	0.007 (0.006)	−0.032 (0.028)	0.002 (0.007)
NA → SU (within)	−0.075 (0.072)	0.546 (0.192)**	0.042 (0.076)
SU → NA (within)	−0.222 (0.229)	0.755 (0.280)**	0.219 (0.201)
Sensation Seeking			
Intercept Mean	0.576 (0.024)***	0.675 (0.041)***	0.601 (0.020)***
Intercept Variance	0.029 (0.025)	0.031 (0.050)	0.014 (0.038)
Slope Mean	0.004 (0.009)	−0.025 (0.013)*	−0.004 (0.007)
Slope Variance	< 0.001 (0.004)	−0.003 (0.005)	−0.002 (0.004)
SS slope ↔ SU intercept	< 0.001 (0.006)	0.013 (0.019)	0.001 (0.007)
SS intercept ↔ SU slope	0.016 (0.008)*	0.063 (0.024)**	0.025 (0.009)**
SS intercept ↔ SU intercept	0.008 (0.018)	−0.057 (0.077)	−0.002 (0.027)
SS slope ↔ SU slope	−0.001 (0.003)	−0.015 (0.007)*	−0.004 (0.003)
SS → SU (within)	0.212 (0.184)	0.090 (0.416)	0.259 (0.158)
SU → SS (within)	0.060 (0.079)	−0.103 (0.062)	−0.033 (0.053)

*NA* negative affect, *SS* sensation seeking, *SU* substance use

*p < 0.05, **p < 0.01, ***p < 0.001

Results from the two-group sensation seeking-substance use model revealed significant between-person level effects, showing positive associations between higher sensation seeking at age 14 and greater increases in substance use from age 14 to 17 in both the low and high executive function groups. This intercept-to-slope association was not significantly different between the two executive function groups (Wald test *χ*^2^ < 0.001, *df* = 1, *p* = 0.985). Additionally, in the low executive function group only, a significant between-person level negative association between the slope of sensation seeking and the slope of substance use was observed. In this model, the low executive function group showed a significant decrease in sensation seeking (reflected by the significant negative mean of the slope factor), whereas the high executive function group showed non-significant change in sensation seeking over time. The decreasing sensation seeking slope was negatively associated with the increasing substance use slope only among the low executive function group. However, this slope-to-slope association was not significantly different between the two executive function groups (Wald test *χ*^2^ = 3.007, *df* = 1, *p* = 0.083). Thus, the magnitude of the negative slope-to-slope association found in the low executive function group was weak. There were no significant within-person associations in the sensation seeking-substance use model.

## Discussion

Prior work has identified negative affect, sensation seeking, and executive function as biobehavioral markers of addiction (Kwako et al., 2018), and these factors have also been associated with adolescent substance use, which may precede later addiction (Giancola & Mezzich, 2003; Hussong et al., 2011). Nonetheless, the extent to which these factors influence substance use development at the within-person level, between-person level, or both has not previously been systematically examined. In addition, prior research has examined the contributions of negative affect, sensation seeking, and executive function primarily at the between-person level. The present study filled these significant gaps in the literature by examining how negative affect, sensation seeking, and executive function—the three biobehavioral domains of the Addictions Neuroclinical Assessment model—contribute to the individual differences in developmental trajectories of substance use progression throughout adolescence. Understanding bidirectional associations between negative affect/sensation seeking and substance use and moderating roles of executive function that predate clinical levels of substance use is crucial. This insight reveals how problematic organizations of the cognitive and emotional systems can lead to later substance use disorders and addiction, providing essential factors to target in prevention programs (Cicchetti, 2010). Latent curve models with structured residuals examining between- and-within-person bidirectional associations among negative affect and sensation seeking with substance use, at varying levels of executive function were estimated. Results indicated that higher negative affect was associated with higher subsequent substance use and higher substance use was associated with higher subsequent negative affect, for adolescents with lower executive function. Higher sensation seeking at baseline (age 14) was associated with increasing substance use from age 14 to 17, regardless of executive function level.

The present study found significant increases in the mean levels of substance use across adolescence as well as individual differences in the developmental trajectories, replicating prior findings that have identified adolescence as a time of increased substance use and significant individual variability in substance use rates (Johnston et al., 2016). In contrast, the present study found non-significant developmental changes on average in negative affect and sensation seeking. For negative affect, this finding is consistent with prior longitudinal work using community samples of adolescents reporting non-significant developmental trends across adolescence, (Griffith et al., 2021; Weinstein et al., 2007). Based on these findings and prior findings of decreasing negative affect in childhood (e.g., Murphy et al., 1999), developmental changes in negative affect may be stabilized by early adolescence (i.e., before age 14), showing non-significant change during mid-late adolescence (i.e., during ages 14–17). For sensation seeking, the present study found no significant change across time. This finding corresponds with prior work that has identified curvilinear trends of sensation seeking throughout adolescence, with levels increasing during early adolescence before stabilizing during mid-adolescence (i.e., 14–17 years) and subsequently declining throughout young adulthood (Steinberg et al., 2018; Harden & Tucker-Drob, 2011).

In the negative affect-substance use model, significant effects emerged at the within-person level only. Specifically, for the low executive function group only, a bidirectional link between substance use and negative affect was found. Increased negative affect was associated with increased substance use the following year, and increased substance use was associated with subsequent increased negative affect. This finding extends prior longitudinal work showing that elevated negative affect is associated with increased use of alcohol to cope with negative feelings, and that the association between negative affect and substance use is reciprocal such that higher negative affect predicts more substance use and more substance use predicts higher negative affect at the within-person level (Mason et al., 2009; Measelle et al., 2006). This association was present only for individuals with lower executive function, and primarily at the within-person level, underscoring the role of executive function in the negative affect to substance use link. Additionally, the significant bidirectional association aligns with past findings and theoretical models suggesting that high levels of negative affect lead to higher risk of drug use as a coping mechanism (Hogarth, 2020) and that individuals continue using substances to avoid higher negative affect (Kassel et al., 2007). Alternatively, these findings suggest that continued substance use increases subsequent negative affect in adolescents. Additionally, these findings support the neurobiological disease model of substance use, which emphasizes that with the progression of substance use, the role of pleasure-seeking changes (Koob & Moal, 1997; Volkow, 2005). The present findings expand upon this theoretical perspective and provide evidence for the reciprocal nature of the negative affect-substance use link. Prior studies on between-person associations between negative affect and substance use show evidence for this negative affect to substance use path (Bradley et al., 2011; Measelle et al., 2006), whereas studies of *daily* within-person associations document no significant effect of negative affect on substance use (e.g., alcohol; Dora et al., 2023). The present study results suggest that the negative affect-substance use year-to-year association is bidirectional at the within-person level, suggesting a more distal (i.e., yearly) cyclical pattern of negative affect and substance use, particularly among low executive function adolescents.

These findings highlight the important role of negative affect in substance use progression and provide support for the internalizing pathway to substance use. During adolescence, evidence for an internalizing pathway to substance use seems to be less prominent than evidence for an externalizing pathway to substance use in the literature, however the prior studies examined these associations solely at the between-person level (Hussong et al., 2017; Rothenberg et al., 2020). It may not be that those who are higher in negative affect *on average* use substances more often. Rather, these data suggest that this pathway is stronger at the within-person level, such that an individual’s elevated negative affect relates to increased substance use, particularly for those with low executive function. Importantly, neuroimaging work has identified key brain regions implicated in impaired regulation of negative affect. Specifically, the amygdala, insula, and prefrontal cortex (PFC) are involved in the regulation and processing of emotions (Wager et al., 2008), and weak resting state connectivity between the amygdala, insula, and regulatory regions of the brain have been shown in individuals with substance use disorders (Wilcox et al., 2016). Collectively, neuroimaging work and the present findings converge to indicate the internalizing pathway to substance use at both the neural and behavioral levels.

An important contribution of the present study is the examination of the role of executive function in adolescent substance use. Research has shown that executive function and related constructs (i.e., cognitive control) moderate the effects of negative affect. For example, compared to individuals with higher executive function, individuals with lower executive function are more likely to ruminate and focus on negative life events, which is associated with elevated depressive symptoms (Gotlib & Joormann, 2010). In the present study, the effect of negative affect was present only among the adolescents with low executive function who may lack the necessary regulatory skills to cope with higher than usual negative affect, and therefore may be more likely to escalate substance use to assuage negative feelings. This finding expands upon the existing literature, demonstrating that executive function may be a protective factor in the internalizing pathway to substance use (i.e., in the presence of negative affect). As such, our findings underscore that negative affect heightens adolescents’ vulnerability to substance use progression, and executive function—a modifiable individual difference factor (Diamond & Ling, 2020)—attenuates this vulnerability.

Turning to the models for sensation seeking and substance use, at the between-person level, higher sensation seeking on average at age 14 was associated with steeper increases in substance use from 14–17 regardless of executive function levels. This finding replicates prior research that identified positive between-person associations between sensation seeking and substance use during adolescence and is in line with the externalizing pathway to substance use (Colder et al., 2013). However, these findings may not provide direct support for the externalizing pathway, given the focus on sensation seeking and the lack of direct measurement of externalizing behaviors. At the within-person level, there were no associations between substance use and sensation seeking. Furthermore, there was no evidence that executive function moderated the sensation seeking-substance use association at the between-person level indicating that high executive function does not necessarily serve as a protective factor against substance use development for adolescents with high sensation seeking. This is consistent with prior research suggesting that sensation seeking and self-control have independent, not interactive, effects on risk taking (Duell et al., 2016). These findings offer important insights into understanding the etiology of substance use during adolescence and identifying youth and adolescents who may benefit the most from substance use prevention programs. That is, those who show high sensation seeking during early adolescence are at the highest risk of developing substance use problems, regardless of executive function level. Conversely, there is no evidence indicating initial substance use as a risk factor for sensation seeking development. These findings have important intervention implications by suggesting that substance use may be prevented via early interventions targeting at-risk adolescents with high sensation seeking. Indeed, prior work has shown that personality-targeted interventions are effective in reducing substance use during adolescence in school-based settings (i.e., Conrod, 2016) and community-based settings (i.e., Edalati & Conrod, 2019). Given that the findings from the present study suggest the association between sensation seeking and substance use during adolescence is not moderated by executive function, interventions targeting executive function may not be most effective for youth high in sensation seeking. Instead, youth with high sensation seeking may benefit more from substance use prevention interventions such as motivational interviewing, which effectively reduces substance use by targeting reward sensitivity and motivation for change (Barnett et al., 2012). Given that adolescent sensation seeking can promote healthy adjustment in certain contexts (Duell & Steinberg, 2020), in addition to reducing high levels of sensation seeking, interventions should aim to redirect sensation seeking from potentially detrimental risk taking (e.g., substance use) to positive risk taking (e.g., motivation for social justice). The present study points to the importance of sensation seeking for substance use particularly at the between-person level during adolescence, which can help inform the refinement of interventions to effectively reduce adolescent substance use.

The slope of sensation seeking was negatively associated with the slope of substance use for the low executive function group only. This may be interpreted in light of the direction of change in sensation seeking growth parameters for each group, and with caution given that the difference in the magnitude of this effect was not statistically significant between the two executive function groups. The high executive function group did not show significant change in the sensation seeking trajectory, whereas the low executive function group showed significant decreases in sensation seeking. Both groups showed significant increases in substance use. These data indicate that faster decreases in sensation seeking are related to slower increases in substance use among adolescents with low executive function, suggesting that those with low executive function would benefit from reducing sensation seeking concerning substance use progression during adolescence.

The present study has several limitations. First, despite the longitudinal study design, causality cannot be inferred due to the correlational nature of the data. Second, measures of negative affect, sensation seeking, and substance use were self-reported by adolescents, which could have introduced method variance and response bias. However, adolescents have been shown to be reliable reporters of their own internal states and sensation seeking behaviors (Huebner & Dew, 1995) and self-report measures are particularly revealing for behaviors that are related to internal experiences (Kendall et al., 1989). Nevertheless, future work should consider utilizing multiple methods (e.g., neurobiological markers, behavioral performance, observations, other informants) to measure those Addictions Neuroclinical Assessment domains to gain a more comprehensive understanding of pathways leading to problematic substance use and addiction. Relatedly, negative affect was assessed annually, yet can be highly labile across days and moments (Naim et al., 2022). Thus, future work may consider this process on a shorter timescale (i.e., across weeks or months) for a more comprehensive understanding of the role of negative affect in substance use. Third, executive function was dichotomized into two groups (i.e., low versus high) for the moderation analysis, and there are concerns regarding dichotomizing continuous variables due to simplifying variance (e.g., MacCallum et al., 2002). We chose the multiple group structural equation modeling approach (1) to decrease model complexity given sample size, and (2) because it allowed us to systematically test where moderating effects of executive function were significant by imposing equality constraints one path at a time and examining changes in model fit using indices that were not influenced by sample size. Supplemental sensitivity analyses suggest that individuals at the extreme low-end of executive function may be particularly at-risk, but that associations between negative affect/sensation seeking, and substance use vary based on how executive function is grouped. Thus, future research with substantially larger sample sizes and more time points should consider examining interaction effects using executive function as a continuous variable to better understand how executive function moderates these associations at varying levels and test whether its moderating effects may change with development. Lastly, for the negative affect and sensation seeking measures, reliability estimates were somewhat low. However, low reliability leads to underestimates of effects, meaning the effects found may be stronger if the negative affect and sensation seeking measures had better reliability (Furr & Bacharach, 2008).

Despite these limitations, this investigation addressed several gaps in the literature. First, the present study included annual assessments of negative affect, sensation seeking, and substance use across adolescence. This is a critical developmental period for substance use initiation and neurobiological development in brain regions involved in reward/incentive sensitivity, emotional reactivity, and executive function. Examining developmental changes in negative affect, sensation seeking, and substance use during this time period allows for a deeper understanding of how the processes that may lead to substance use progression unfold. Second, the socioeconomically diverse sample includes adolescents in rural, suburban and urban settings from understudied and underserved communities that include multiple economically distressed counties, towns, and cities. These regions have current and historically high rates of substance use and addiction in comparison with the rest of the nation (Appalachian Regional Commission, 2023). Understanding the effects of biobehavioral markers of addiction on prospective development of substance use in this sample advances the theoretical understanding of risk and protective factors on substance use development among at-risk youth. Third, the present study utilized a multivariate LCM-SR, which facilitated the ability to disaggregate between-person and within-person processes by which negative affect, sensation seeking, and substance use change over time. Examining changes within individuals across time (i.e., how an individual moves from one state in time to the next on one dimension, depending on their state on another dimension) is directly relevant to intervention efforts. Understanding within-person changes helps identify which psychological domains to target and which can engender subsequent changes in symptoms. Further, examining the between-person effects while accounting for within-person effects clarifies the between-person differences in negative affect and sensation seeking related to substance use, and informs who may be at elevated risk for substance use progression, enabling targeted preventive intervention.

## Conclusion

Past research suggests that sensation seeking and negative affect are risk factors for adolescent substance use. However, the bidirectional associations between these risk factors and substance use at both the between-and within-person levels have not been clearly understood. The present study illustrates the between- and within-person level processes through which emotional risk (incentive salience and negative affect) and cognitive protective (executive function) factors interface to contribute to substance use throughout adolescence. At the within-person level, higher levels of negative affect one year were associated with more substance use the following year, and higher levels of substance use were associated with higher subsequent negative affect. Further, executive function served as a protective factor in this association such that these bidirectional within-person associations between negative affect and substance use were significant only for adolescents with low executive function. These findings help clarify the mixed findings in the prior literature on the internalizing pathway to substance use and offer important implications for prevention. The internalizing pathway to substance use may be weakened by enhancing adolescent executive function, which can harness the reciprocal within-person processes between negative affect and substance use. At the between-person level, individuals higher in sensation seeking showed steeper increases in substance use from age 14 to 17 regardless of their executive function. These findings contribute to the literature on adolescent risk factors for substance use, emphasizing the significant role of high sensation seeking during early adolescence in increasing substance use trajectories across adolescence.

## Supplementary information

Supplementary Information
