# Supplementary material for: Negative Affect, Sensation Seeking, and Adolescent Substance Use Development: The Moderating Role of Executive Function
Source: J Youth Adolesc. 2024 Aug 10;53(11):2654–68. doi: 10.1007/s10964-024-02065-9 (PMC11467108; doi:10.1007/s10964-024-02065-9)
Supplement: Supplementary file 1 — Supplementary Information [file 10964_2024_2065_MOESM1_ESM.docx]

**Supplemental Materials**

**Negative Affect, Sensation Seeking, and Adolescent Substance Use Development:**

**The Moderating Role of Executive Function**

Ann Folker, Kristin M. Peviani, Kirby Deater-Deckard, Warren K. Bickel,

Laurence Steinberg, Brooks Casas, & Jungmeen Kim-Spoon

**Table S1.**

*Latent Growth Factor Correlations and Cross-Lagged Regression Coefficients between Negative Affect/Sensation Seeking and Substance Use at High and Low Executive Function Controlling for Income, Race, and Sex*

|  | High EF | Low EF | Full Sample |
| --- | --- | --- | --- |
|  | *b (SE)* | *b (SE)* | *b (SE)* |
| Negative Affect |  |  |  |
| NA slope ↔ SU intercept | -0.009 (0.010) | 0.021 (0.069) | -0.008 (0.013) |
| NA intercept ↔ SU slope | 0.014 (0.016) | -0.024 (0.070) | -0.002 (0.018) |
| NA intercept ↔ SU intercept | 0.039 (0.026) | 0.017 (0.273) | 0.049 (0.034) |
| NA slope ↔ SU slope | 0.007 (0.006) | -0.020 (0.025) | 0.005 (0.006) |
| NA → SU (within) | -0.081 (0.072) | 0.672 (0.177)*** | 0.039 (0.081) |
| SU → NA (within) | -0.237 (0.228) | 0.739 (0.249)*** | 0.226 (0.189) |
| Sex → NA intercept | 0.302 (0.114)** | -0.098 (0.085)** | 0.320 (0.103)** |
| Race → NA intercept | 0.005 (0.152) | 0.236 (0.223) | 0.051 (0.127) |
| ITN → NA intercept | 0.019 (0.029) | 0.083 (0.072) | 0.017 (0.027) |
| Sex → NA slope | -0.006 (0.044) | -0.098 (0.085) | -0.013 (0.040) |
| Race → NA slope | -0.052 (0.060) | -0.179 (0.087)* | -0.077 (0.050) |
| ITN → NA slope | -0.006 (0.012) | -0.031 (0.028) | -0.021 (0.016) |
| Sex → SU intercept | -0.084 (0.064) | -0.014 (0.148) | -0.055 (0.060) |
| Race → SU intercept | 0.140 (0.085) | 0.026 (0.155) | 0.113 (0.075) |
| ITN → SU intercept | -0.008 (0.016) | -0.067 (0.052) | -0.021 (0.016) |
| Sex → SU slope | 0.013 (0.047) | 0.019 (0.092) | 0.014 (0.043) |
| Race → SU slope | < 0.001 (0.063) | 0.157 (0.098) | 0.044 (0.053) |
| ITN → SU slope | -0.002 (0.012) | 0.013 (0.031) | -0.004 (0.012) |
| Sensation Seeking |  |  |  |
| SS slope ↔ SU intercept | -0.001 (0.005) | 0.020 (0.026) | 0.002 (0.008) |
| SS intercept ↔ SU slope | 0.017 (0.008)* | 0.074 (0.030)* | 0.024 (0.009)* |
| SS intercept ↔ SU intercept | 0.007 (0.017) | -0.090 (0.120) | -0.006 (0.032) |
| SS slope ↔ SU slope | -0.001 (0.003) | -0.016 (0.008)* | -0.004 (0.003) |
| SS → SU (within) | 0.185 (0.185) | 0.119 (0.453) | 0.286 (0.156) |
| SU → SS (within) | 0.059 (0.080) | -0.107 (0.071) | -0.022 (0.052) |
| Sex → SS intercept | 0.042 (0.046) | -0.049 (0.084) | 0.018 (0.040) |
| Race → SS intercept | 0.102 (0.062) | 0.029 (0.093) | 0.092 (0.050) |
| ITN → SS intercept | -0.007 (0.012) | 0.021 (0.028) | -0.005 (0.011) |
| Sex → SS slope | -0.033 (0.016)* | -0.041 (0.025) | -0.033 (0.014)* |
| Race → SS slope | -0.017 (0.023) | -0.028 (0.027) | -0.022 (0.018) |
| ITN → SS slope | < 0.001 (0.004) | -0.002 (0.008) | < 0.001 (0.004) |
| Sex → SU intercept | -0.084 (0.064) | 0.011 (0.137) | -0.053 (0.059) |
| Race → SU intercept | 0.136 (0.086) | 0.015 (0.153) | 0.103 (0.074) |
| ITN → SU intercept | -0.010 (0.016) | -0.058 (0.046) | -0.024 (0.016) |
| Sex → SU slope | 0.008 (0.046) | -0.025 (0.092) | 0.005 (0.043) |
| Race → SU slope | 0.006 (0.063) | 0.179 (0.099) | 0.052 (0.053) |
| ITN → SU slope | 0.001 (0.012) | -0.014 (0.030) | < 0.001 (0.012) |

*Note*. EF = executive function; NA = negative affect; SS = sensation seeking; SU = substance use; Sex = binary adolescent sex (0 = male, 1 = female); Race = binary adolescent race (0 = White, 1 = Other race); ITN = income-to-needs ratio at Time 1.

**p* < .05, ** *p* < .01, ****p* < .001.

**Table S2.**

*Latent Growth Factor Correlations and Cross-Lagged Regression Coefficients between Negative Affect/Sensation Seeking and Substance Use at High and Low Executive Function (median split- 50% low/50% high)*

|  | High EF | Low EF |
| --- | --- | --- |
|  | *b (SE)* | *b (SE)* |
| Negative Affect |  |  |
| Intercept Mean | 1.837 (0.068)*** | 2.000 (0.082)*** |
| Intercept Variance | 0.246 (0.067)*** | 0.291 (0.208) |
| Slope Mean | 0.046 (0.026) | 0.014 (0.031) |
| Slope Variance | 0.006 (0.016) | -0.011 (0.036) |
| NA slope ↔ SU intercept | -0.019 (0.015) | 0.015 (0.024) |
| NA intercept ↔ SU slope | 0.022 (0.021) | -0.016 (0.031) |
| NA intercept ↔ SU intercept | 0.041 (0.040) | -0.002 (0.066) |
| NA slope ↔ SU slope | 0.006 (0.007) | 0.002 (0.013) |
| NA → SU (within) | -0.104 (0.089) | 0.144 (0.134) |
| SU → NA (within) | 0.057 (0.301) | 0.179 (0.248) |
| Sensation Seeking |  |  |
| Intercept Mean | 0.591 (0.028)*** | 0.613 (0.030)*** |
| Intercept Variance | 0.034 (0.022) | 0.048 (0.039) |
| Slope Mean | 0.001 (0.011) | -0.008 (0.009) |
| Slope Variance | 0.003 (0.003) | 0.055 (0.017)** |
| SS slope ↔ SU intercept | -0.006 (0.008) | < 0.001 (0.009) |
| SS intercept ↔ SU slope | 0.006 (0.011) | 0.039 (0.014)** |
| SS intercept ↔ SU intercept | 0.023 (0.030) | 0.007 (0.032) |
| SS slope ↔ SU slope | -0.001 (0.003) | -0.004 (0.005) |
| SS → SU (within) | 0.219 (0.204) | 0.122 (0.330) |
| SU → SS (within) | 0.079 (0.095) | -0.125 (0.074) |

*Note*. EF = executive function; NA = negative affect; SS = sensation seeking; SU = substance use.

** *p* < .01, ****p* < .001.
